# *Begonia jinyunensis* (Begoniaceae, section *Platycentrum*), a new palmately compound leaved species from Chongqing, China

**DOI:** 10.1186/s40529-014-0062-6

**Published:** 2014-08-02

**Authors:** Bo Ding, Koh Nakamura, Yoshiko Kono, Meng-Jung Ho, Ching-I Peng

**Affiliations:** 1 College of Life Science and Engineering, Chongqing Three Gorges University, Chongqing, 404000 China; 2 Herbarium (HAST), Biodiversity Research Center, Academia Sinica, Taipei 11542 Taiwan

**Keywords:** Begonia jinyunensis, Begonia hemsleyana, Begoniaceae, Chromosome number, Flora of China, ITS, Limestone, New species, rpl16, Sect. Platycentrum

## Abstract

**Background:**

Continental China is the center of *Begonia* species diversity in Asia and contains more than 60 species out of about 110 named species of section *Platycentrum*. Mt. Jinyun, located in Chongqing City at the upper reaches of the Yangtze River, harbors a subtropical broadleaved forest with high species diversity. During a botanical survey in Mt. Jinyun, an unknown *Begonia* species of sect. *Platycentrum* with palmately compound leaves was collected and studied based on detailed morphological observations and cytological and molecular phylogenetic analyses.

**Results:**

The unknown *Begonia* bears a superficial resemblance to *B. hemsleyana* in having palmately compound leaves, a feature unseen in other species of sect. *Platycentrum* in China. It is however sharply distinct from the latter in the acaulous habit with aerial stems seen only at anthesis and long rhizomes (vs. erect stems to 70 cm or taller with short rhizomes), 4–6 pinnatilobed leaflets with indistinct, decurrent petiolules (vs. 7–10 serrate leaflets with distinct petiolules), and white (vs. pink) tepals. Molecular phylogenetic analyses based on nuclear ribosomal DNA and chloroplast DNA sequences indicated that this species was allied to *Platycentrum* species occurring in Southwest and South-central China and Vietnam, including *B. hemsleyana*, and clearly separable from these species. Somatic chromosome number of 2*n* = 22 was reported for this unknown species. The diploid chromosome number is agreeable with those published for *Begonia* sect. *Platycentrum*.

**Conclusions:**

The unknown *Begonia* is confirmed to be a new species of sect. *Platycentrum* and hereto described as *Begonia jinyunensis* C.-I Peng, B. Ding & Q. Wang.

**Electronic supplementary material:**

The online version of this article (doi:10.1186/s40529-014-0062-6) contains supplementary material, which is available to authorized users.

## Background

The mega-diverse genus *Begonia* L. (Begoniaceae) comprises ca. 1,500–1,600 species widely distributed in the tropical and subtropical regions of the world except Australia (Kiew [2005], Tebbitt [2005], Chung et al. [2014]). Section *Platycentrum* (Klotzsch) A. DC. comprises about 110 species of herbs and subshrubs that are widely distributed in Asia, ranging from India to the Himalayas, Indo-China, China, Taiwan, and Malesia (Doorenbos et al. [1998], Shui et al. [2002]). Continental China is the center of *Begonia* species diversity in Asia and contains more than 60 *Platycentrum* species predominantly in South of Yangtze River (Shui et al. [2002], Gu [2007], Wei et al. [2007]). They are either terrestrial on thick humus, epiphytic on trees, or epipetric on rock faces or crevices at 100–2,200 m altitude (Shui et al. [2002]). Many *Begonia* species are local- or site-endemics and even after the publication of Flora of China (Gu et al. [2007]) many new *Begonia* species have been discovered (Liu et al. [2007], Shui [2007], Wei et al. [2007], Ku et al. [2008], Li et al. [2008], Peng et al. [2008a], [2008b], [2010], [2012], [2013], [2014]), suggesting that the species-rich genus has not been fully explored in China.

**Figure 1:**
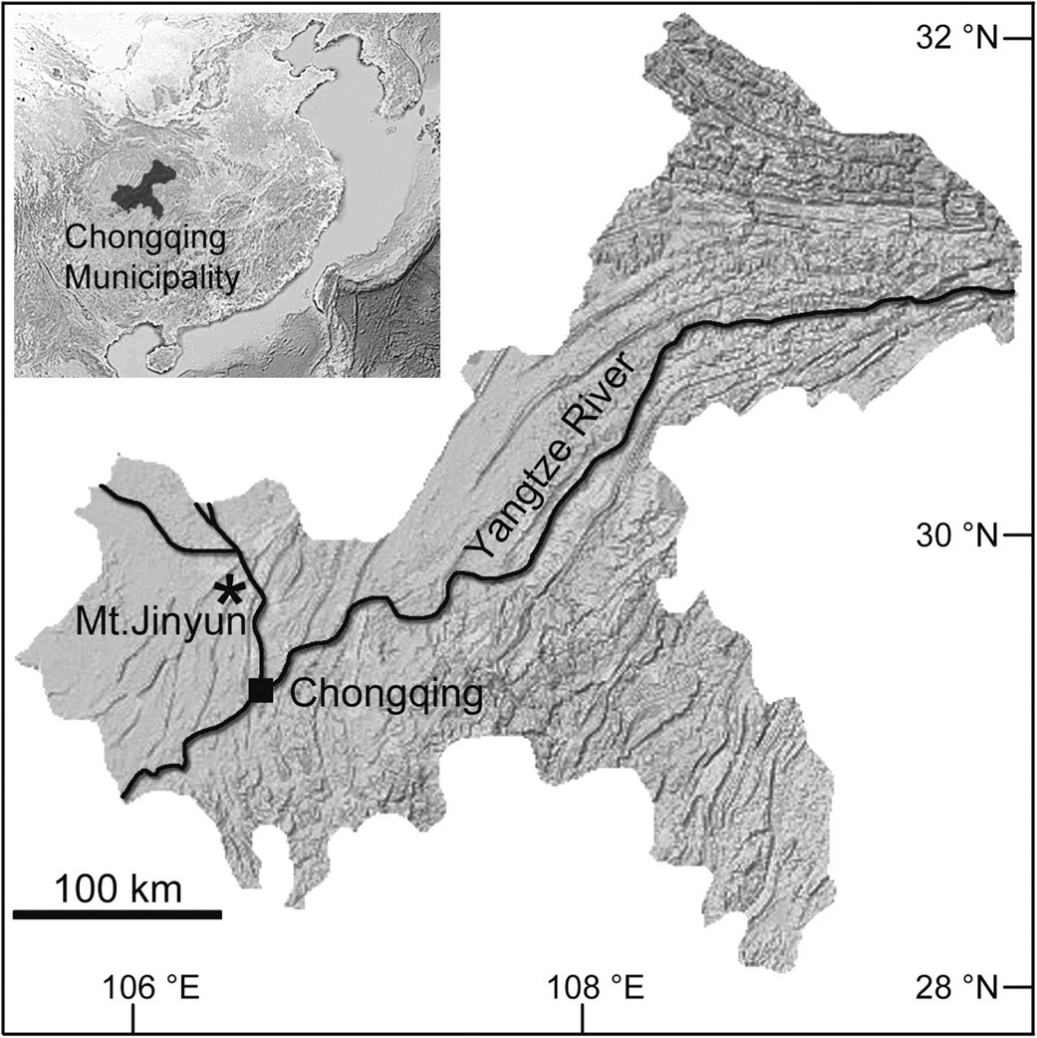
**Distribution of** ***Begonia jinyunensis*** **(Mt. Jinyun of Chongqing Municipality, Southwest China).**

Mt. Jinyun (29° 49' N, 106° 20' E; Figure 1) is located at the upper reaches of the Yangtze River and has the highest peak (950 m) in Chongqing Municipality, Southwest China. The climate is subtropical monsoon, with annual mean temperature of 18.3°C and annual rainfall of 1,105 mm, and the vegetation is mixed evergreen broad-leaved and conifer forest, with patches of bamboos (Qing et al. [2004]). The area, although only 20 km away from the center of the megacity, harbors a typical forest landscape of the Yangtze River basin and high species diversity, and is protected as Jinyun Mountain National Nature Reserve with the core area of 1,235 hectares (Qing et al. [2004]). In a botanical survey of the nature reserve in July 2013, Bo Ding, Qian Wang, and Hai-Jun Wen collected an unknown *Begonia* of sect. *Platycentrum* with palmately compound leaves and sent it to Ching-I Peng for cultivation at the experimental greenhouse in Academia Sinica for careful examination. In *Begonia* sect. *Platycentrum*, only one species, *B. hemsleyana* Hook. f. occurring in Southern China (Southwest Guangxi and Southeast Yunnan) and northern Vietnam (Gu et al. [2007]), has palmately compound leaves. Based on detailed morphological study, both of herbarium materials and living collection, and cytological and molecular phylogenetic analyses, we confirmed that the unknown *Begonia* is a new species, which is hereby described as *Begonia jinyunensis* C.-I Peng, B. Ding & Q. Wang.

## Methods

### Morphological study

Living collections of *Begonia jinyunensis* (*Bo Ding 20130701*, *Ching-I Peng 24108*, *24109*, *24110*) were used for morphological observations. In addition, herbarium specimens representative of *B. jinyunensis* were carefully studied in IBSC.

### Molecular phylogenetic analyses

Molecular phylogenetic analyses of *B. jinyunensis* incorporated *B. hemsleyana* and 21 species (Appendix 1); these 21 species fell in the same clade with *B. hemsleyana* in a previous phylogenetic study on Asian *Begonia* based on concatenated sequences of the internal transcribed spacer (ITS) region (including ITS1 and ITS2 and the 5.8S rRNA gene) of 18S-26S nuclear ribosomal DNA (nrDNA) and an intron of rpl16 gene (rpl16) of chloroplast DNA (cpDNA) (Chung et al. [2014]). These species were mostly sect. *Platycentrum* (14 species including *B. hemsleyana*) but also included five species of sect. *Diploclinium* (Lind.) A. DC., two of sect. *Sphenanthera* (Hassk.) Warb., and *B. longicarpa* K. Y. Guan & D. K. Tian, currently not assigned to any section (Chung et al., [2014]). Note that Asian sections of *Begonia* are largely non-monophyletic (Thomas et al. [2011], Chung et al. [2014]). Outgroup taxa were *B. parvula* H. Lév. & Vaniot of sect. *Reichenheimea* (Klotzsch) A. DC. and *B. ravenii* C.-I Peng & Y. K. Chen of sect. *Diploclinium*, which were placed outside of the clade (Chung et al. [2014]). For *B. jinyunensis* three plants (*Ching-I Peng 24108*, *24109*, *24110*) from three localities in Mt. Jinyun were examined; for *B. hemsleyana* four plants, three from Yunnan (*Ching-I Peng 17590*, *20933*, *22778*) and one from Vietnam (*Ching-I Peng 20204*), were studied. For the other species one sample each was used. Samples sequenced in this study are indicated with asterisks (Appendix 1) and sequences of the other samples were cited from Chung et al. ([2014]). Methods of DNA extraction, PCR, and DNA sequencing followed Nakamura et al. ([2012]). DNA sequences were aligned using ClustalX ver. 1.8 (Thompson et al. [1997]) and then manually adjusted. Phylogenetic analyses were based on a Bayesian approach using MrBayes ver. 3.1.2 (Ronquist & Huelsenbeck [2003]) and a maximum parsimony (MP) criterion using PAUP* ver. 4.0b10 (Swofford [2002]).

In the Bayesian phylogenetic analysis, the best substitution models for the ITS and rpl16 data were selected as SYM + I and GTR + I, respectively, using KAKUSAN4 (Tanabe [2011]) based on Bayesian information criterion (BIC). Two separate runs of Metropolis coupled Markov chain Monte Carlo analyses were performed, each with a random starting tree and four chains (one cold and three heated). The chain length was ten million generations, and the chain was sampled every one thousandth generation from the cold chain. The mixing and convergence of the chains of the two runs was assessed using Tracer ver. 1.5.0 (Drummond & Rambaut [2007]). The first 10% of the total 10,000 sample trees were discarded as burn-in. After the burn-in, the effective sample sizes of all parameters were > 200, indicating that the analyses sampled the posterior distributions of each parameter satisfactorily, and the values of Average Standard Deviation of Split Frequency (ASDSF) were below 0.005. The 50% majority rule consensus tree of all the post-burn-in trees with Bayesian posterior probabilities (*PP*) was visualized with FigTree ver. 1.3.1 (Drummond & Rambaut [2007]).

In the MP phylogenetic analysis, indels were treated as missing data. The characters were treated as unordered, and the character transformations were equally weighted. The branch collapse option was set to collapse at a minimum length of zero. A heuristic parsimony search was performed with 1,000 replicates of random additions of sequences with ACCTRAN character optimization, tree bisection*–* reconnection (TBR) branch swapping, and MULTREES and STEEPEST DESCENT options on. Statistical support for each clade was assessed by bootstrap analysis (Felsenstein [1985]). Ten thousand replicates of heuristic searches, with the TBR branch swapping switched on and MULTREES options off, were performed to calculate bootstrap percentages (*BP*).

### Chromosome cytology

Somatic chromosome morphology was studied for one plant of *B. jinyunensis* (*Ching-I Peng 24110*, HAST) using root tips. The methods of pretreatment, fixation, and staining for chromosome observations followed our previous paper (Peng et al. [2012]). Classification of the chromosome complements based on centromere position at mitotic metaphase followed Levan et al. ([1964]).

## Results and discussion

### Molecular phylogeny based on nrDNA and cpDNA

**Figure 2:**
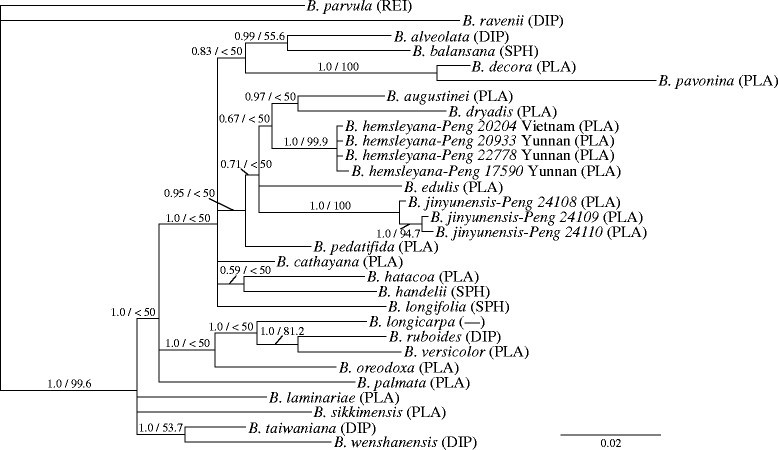
**Bayesian majority-rule consensus tree with mean branch length based nrITS plus cpDNA rpl16 intron sequences.** The numerals on branches are Bayesian posterior probabilities (*PP*, *left*) and bootstrap percentages (*BP*, *right*) in the MP analysis. Section designation is indicated in parentheses (*Diploclinium*–DIP; *Platycentrum*–PLA; *Reichenheimia*–REI; *Sphenanthera*–SPH). Scale bar shows the number of expected substitutions per site.

The aligned length of the ITS and rpl16 sequences was 695 bp and 1,002 bp, respectively. In the concatenated data set, 323 nucleotide substitutions were found in 277 variable sites and 120 sites were parsimony informative among them. The Bayesian 50% majority rule consensus tree with mean branch length and *PP* is depicted (Figure 2). The MP analysis yielded 44 equally parsimonious trees of 641 steps with a consistency index (CI) = 0.682, a retention index (RI) = 0.614, and a rescaled consistency index (RC) = 0.418. Clades supported with *BP* ≥ 50% in the MP strict consensus tree (not shown) were all recognized in the Bayesian tree. *BP* was plotted on the Bayesian tree. Only clades supported by *PP* ≥ 0.95 and/or *BP* ≥ 70% were considered adequately supported. *Begonia jinyunensis* resided in a clade with *B. hemsleyana*, *B. augustinei*, *B. dryadis*, *B. edulis*, and *B. pedatifida* (*PP* = 0.95/*BP* < 50%). These are species of sect. *Platycentrum* in Southwest and South-central China and Vietnam (Gu et al. [2007]). Although phylogenetic relationship among the species within this clade was not fully resolved except for the clade of *B. augustinei* and *B. dryadis* (0.97/< 50%), the six species were clearly separated from each other, and *B. jinyunensis* (1.0/100%) and *B. hemsleyana* (1.0/99%) were reciprocally monophyletic.

### Chromosomal features

**Figure 3:**
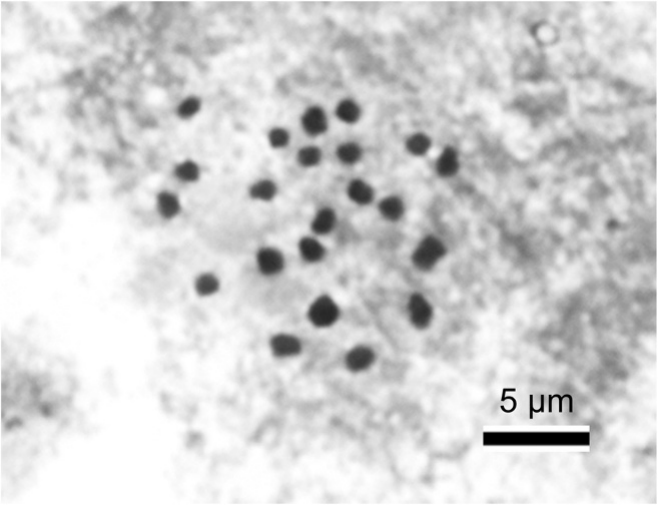
**Somatic chromosomes at metaphase of** ***Begonia jinyunensis*** **(2** ***n*** **= 22,** ***Ching-I Peng*** **24110, HAST).**

Somatic chromosomes at metaphase of *B. jinyunensis* were determined to be 2*n* = 22 (Figure 3). The 22 chromosomes gradually varied in length from ca. 1.0 to 1.8 μm. Several larger chromosomes had centromeres at median or submedian positions but centromere position of smaller chromosomes could not be determined. No satellite was observed. For *B. hemsleyana*, somatic chromosome number 2*n* = 22 was reported for plants from Yunnan, China (Tian et al. [2002]. Note that the number was mistyped as 2*n* = 20 in the abstract and thereby erroneously cited in Index to Plant Chromosome Numbers [http://www.tropicos.org/Project/IPCN]; Nakata et al. [2003]). *Begonia jinyunensis* and *B. hemsleyana* were karyologically indistinguishable due to the small chromosome size.

### Taxonomic treatment

#### Begonia jinyunensis

**Figure 4:**
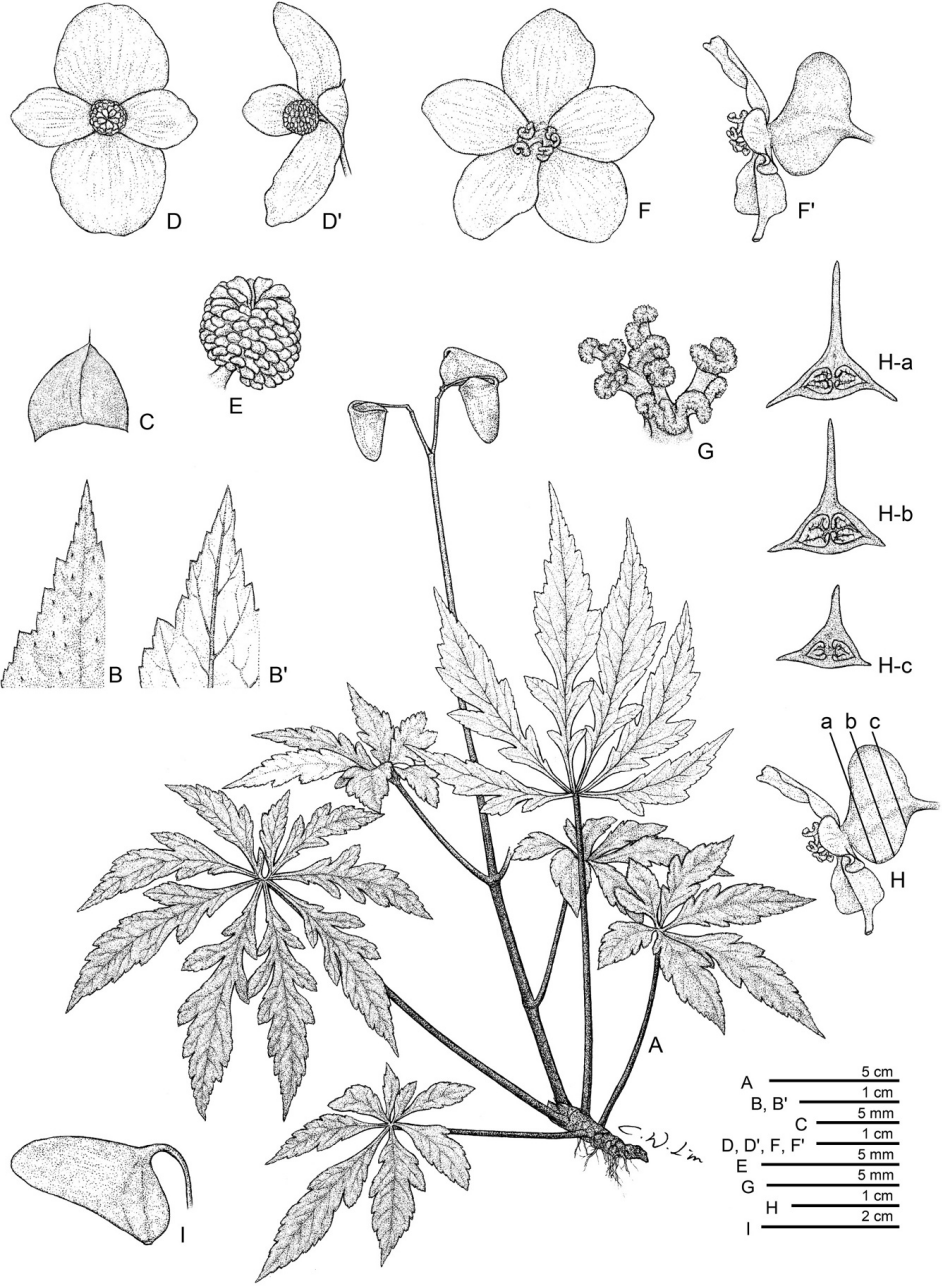
***Begonia jinyunensis*** **C.-I Peng, B. Ding & Q. Wang. A**, Habit; **B**, Leaf adaxial surface; B', Leaf abaxial surface; **C**, Stipule; **D**, Staminate flower, ventral view; D', Staminate flower, lateral view; **E**, Stamens; **F**, Pistillate flower, ventral view; F', Pistillate flower, lateral view; **G**, Styles and stigmas; **H**-a,b,c, Transverse sections of developing capsule; **I**, Capsule. [Drawn from *Ching-I Peng 24108, 24109, 24110* (HAST)].

**Figure 5:**
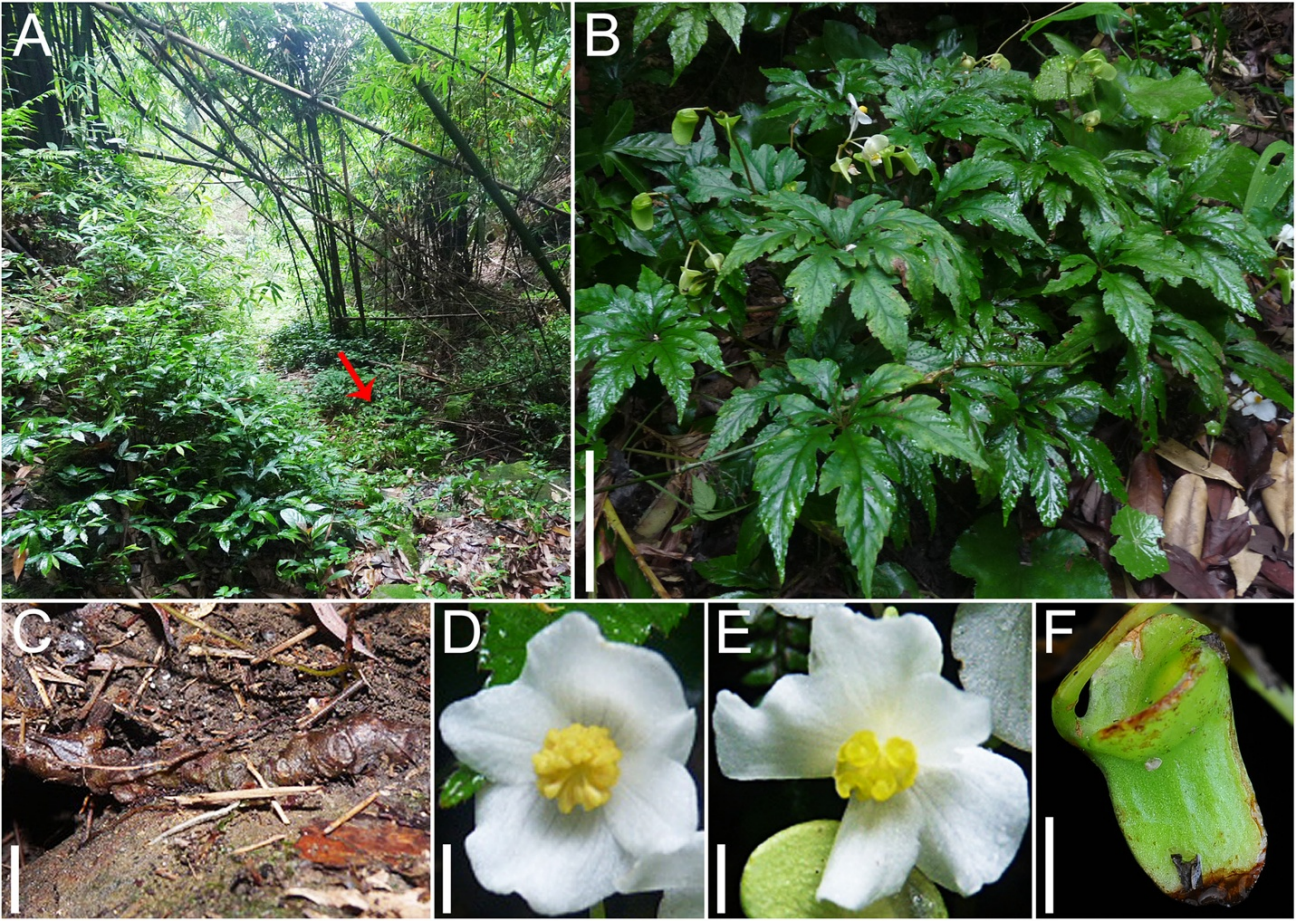
***Begonia jinyunensis*** **C.-I Peng, B. Ding & Q. Wang. A**, Habitat (*B. jinyunensis* indicated by an arrow); **B**, Habit; **C**, Rhizome; **D**, Staminate flower; **E**, Pistillate flower; **F**, Capsule. Scale bars are 5 cm for **B**, 1 cm for **C** and **F**, 5 mm for **D** and **E**. [**A** and **C** from *Ching-I Peng 24109* (HAST); **B**, **D**, **E**, and **F** from *Ching-I Peng 24108* (HAST)].

C.-I Peng, B. Ding & Q. Wang, *sp. nov.* (sect. *Platycentrum*) (Figures 4 and 5).

Type:—CHINA. Chongqing Municipality, Beibei, Mt. Jinyun, Jinyun Mountain National Nature Reserve, 29° 49' N, 106° 20' E, ca. 789 m alt., 10 July 2013, *Bo Ding 20130701*, with Qian Wang and Hai-Jun Wen (holotype, HAST; isotype, CDBI, E, IBSC, KUN, PE) 縉雲秋海棠.

#### Diagnosis

*Begonia jinyunensis* bears a superficial resemblance to *B. hemsleyana* in having palmately compound leaves, a feature unseen in other species of sect. *Platycentrum* in China. It is however sharply distinct from the latter in the acaulous habit with short aerial stems seen only at anthesis and long rhizomes (vs. erect stems to 70 cm or taller with short rhizomes), 4–6 pinnatilobed leaflets with indistinct, decurrent petiolules (vs. 7–10 serrate leaflets with distinct petiolules), and white (vs. pink) tepals.

#### Description

Herbs, monoecious; epipetric; perennial; rhizomatous. Rhizomes reddish-brown, 5–15 cm or longer, 6–16 mm across, subglabrous, internodes 2–12 mm. Stipules reddish-brown, widely ovate, ca. 3–6 × 4–6 mm, slightly keeled, apex shortly aristate, subglabrous, deciduous. Leaves many, arising directly from the rhizome, with 1–3 cauline leaves at anthesis; petioles 10–22 cm long, subglabrous or sparely hispidulous. Blade pale to dark green above, tinged reddish (pronounced on veins) beneath, palmately compound, leaflets 4–6, obliquely oblong-lanceolate or obovate-lanceolate, 4–10 × 1.5–3 cm, abaxially sparsely setulose, adaxially sparsely hirsute, margins pinnatilobed, apex caudate-acuminate or acuminate, base cuneate or attenuate into a short, winged petiolule 2–5 mm long. Inflorescence 10–35 cm long, subglabrous; pedicel 1–4 cm; bracts ovate-oblong, 4–5 × 2–3 mm, apex acute. Staminate flowers: pedicel 11–15 mm, tepals 4, white, outer 2 broadly ovate, nearly as long as wide, 12–15 × 11–15 mm, inner 2 oblong-obovate, 8–10 × 4–6 mm; stamens numerous. Pistillate flowers: pedicel 10–40 mm, tepals 5, white, obovate to broadly obovate, 7–15 × 5–10 mm; ovary 2-loculed; placentae axile, bilamellate; styles 2, ca. 5 mm long, fused to halfway. Capsule nodding, ellipsiod, fruit body ca. 10–20 mm long, 7–12 mm thick (wings excluded), unequally 3-winged; abaxial wing falcate-ligulate, 12–18 mm wide. Somatic chromosome number, 2*n* = 22 (Figure 3).

#### Additional specimens examined

CHINA. Chongqing Municipality. Mt. Jinyun, Pokong Tower, under shady rocks, 780 m alt., 5 August 1934, T.-H. Tu 5087 (IBSC); Mt. Jinyun ("Tsing-yun Shan"), s.d., received by IBSC in May 1940, *L.-Y. Lin 1082* (IBSC); Mt. Jinyun, Jinyun Elementary School, collected and presented by Bo Ding, 29 July, 2013, *Ching-I Peng 24108* (HAST); Mt. Jinyun, Qinglongzhai, collected and presented by Bo Ding, 29 July, 2013, *Ching-I Peng 24109* (HAST); Mt. Jinyun, Shaolongguan, collected and presented by Bo Ding, 29 July, 2013, *Ching-I Peng 24110* (HAST).

#### Distribution, habitat and ecology

*Begonia jinyunensis* is currently known only from Jinyun Mountain National Nature Reserve. The species usually grows on limestone faces of shaded moist environments such as valleys and broadleaved forests. Flowering in July; fruiting in August.

#### Etymology

The specific epithet is derived from the type locality, Mt. Jinyun.

#### IUCN Red list category

Vulnerable (VU D2). *Begonia jinyunensis* is known only from a narrow area of Mt. Jinyun, Chongqing Municipality. Although the area is under protection as a national nature reserve, habitat disturbance brought about by human activities such as tourism and maintenance/building of roads/walking trails may have a negative impact on the species.

## Conclusions

Detailed morphological observations and molecular phylogenetic analyses based on nuclear ribosomal DNA and chloroplast DNA sequences supported the recognition of the new species *Begonia jinyunensis*. Somatic chromosome number of 2*n* = 22 was reported for this new species. *Begonia jinyunensis* is currently known only from the type locality, Mt. Jinyun, Chongqing Municipality, Southwest China.

## Appendix 1

*Begonia* species included in the molecular phylogenetic analyses. Shown here are species name, section name (*Diploclinium*–DIP; *Platycentrum*–PLA; *Reichenheimia*–REI; *Sphenanthera*–SPH), voucher specimen (HAST), and GenBank accession numbers for rpl16 and ITS. Asterisks indicate samples sequenced in this study; sequences of the other samples were cited from Chung et al. ([2014]).

*Begonia alveolata* T. T. Yu, DIP, *Ching-I Peng 20421*, KF707933, AY048977. *Begonia augustinei* Hemsl., PLA, *Ching-I Peng 20759*, KF707936, KF636421. *Begonia balansana* Gagnep., SPH, *Ching-I Peng 21928*, KF707939, AF485091. *Begonia cathayana* Hemsl., PLA, *Ching-I Peng 20288*, KF707948, AF280106. *Begonia decora* Stapf, PLA, *Ching-I Peng 20261*, KF707956, KF636435. *Begonia dryadis* Irmsch., PLA, *Ching-I Peng 18016*, KF707959, KF636436. *Begonia edulis* H. Lév., PLA, *Ching-I Peng 18747*, KF707960, KF636437. *Begonia handelii* Irmsch., SPH, *Ching-I Peng 17513*, KF707969, AY048982. *Begonia hatacoa* Buch.-Ham. ex D. Don, PLA, *Ching-I Peng 20861*, KF707970, KF636444. *Begonia hemsleyana* Hook. f., PLA, *Ching-I Peng 17590*, KF707971, AF485099; Ching-I Peng 20204*, AB972933, AB972927; Ching-I Peng 20933*, AB972934, AB972928; Ching-I Peng 22778*, AB972935, AB972929. *Begonia jinyunensis* C.-I Peng, B. Ding & Q. Wang, PLA, Ching-I Peng 24108*, AB972936, AB972930; Ching-I Peng 24109*, AB972937, AB972931; Ching-I Peng 24110*, AB972938, AB972932. *Begonia laminariae* Irmsch., PLA, *Ching-I Peng 17447*, KF707982, KF636455. *Begonia longicarpa* K. Y. Guan & D. K. Tian, No section designation, *Ching-I Peng 18651*, KF707987, AY048985. *Begonia longifolia* Blume, SPH, *Ching-I Peng 16795*, KF707988, AF485105. *Begonia oreodoxa* Chun & F. Chun ex G. Y. Wu & T. C. Ku, PLA, *Ching-I Peng 20454*, KF707996, KF636467. *Begonia palmata* D. Don, PLA, *Ching-I Peng 20993*, KF707998, KF636468. *Begonia parvula* H. Lév. & Vaniot, REI, *Ching-I Peng 20396*, KF708001, KF636471 *Begonia pavonina* Ridl., PLA, *Ching-I Peng 20239*, KF708002, KF636472. *Begonia pedatifida* H. Lév., PLA, *Ching-I Peng 18779*, KF708003, KF636473. *Begonia ravenii* C.-I Peng & Y. K. Chen, DIP, *Ching-I Peng 14855*, KF708009, KF636479. *Begonia ruboides* C. M. Hu ex C. Y. Wu & T. C. Ku, DIP, *Ching-I Peng 18705*, KF708011, KF636481. *Begonia sikkimensis* A. DC., PLA, *Ching-I Peng 20848*, KF708015, KF636485. *Begonia taiwaniana* Hayata, DIP, *Ching-I Peng 18111*, KF708020, KF636488. *Begonia versicolor* Irmsch., PLA, *Ching-I Peng 18688*, KF708023, AF485090. *Begonia wenshanensis* C. M. Hu ex C. Y. Wu & T. C. Ku, DIP, *Ching-I Peng 20516*, KF708025, AY048974.

## Authors’ original submitted files for images

Below are the links to the authors’ original submitted files for images. Authors’ original file for figure 1 Authors’ original file for figure 2 Authors’ original file for figure 3 Authors’ original file for figure 4 Authors’ original file for figure 5
