# Supplementary figures and images for: Begonia jinyunensis (Begoniaceae, section Platycentrum), a new palmately compound leaved species from Chongqing, China
Source: Bot Stud. 2014 Aug 2;55:62. doi: 10.1186/s40529-014-0062-6 (PMC5430339; doi:10.1186/s40529-014-0062-6)

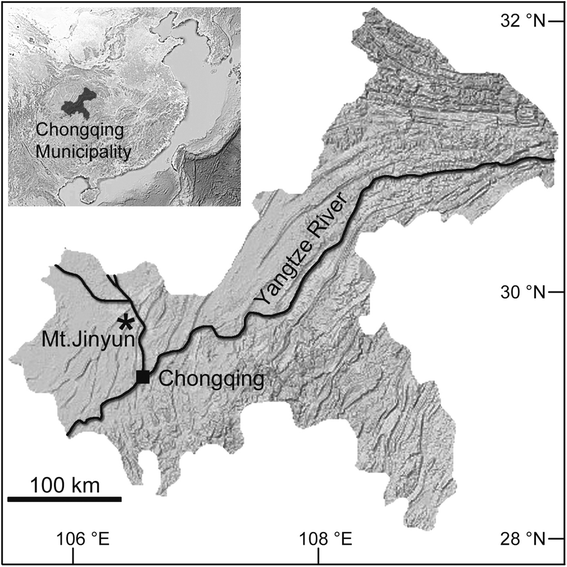

Supplement: Supplementary file 1 — Authors’ original file for figure 1 [file 40529_2014_9062_MOESM1_ESM.gif]

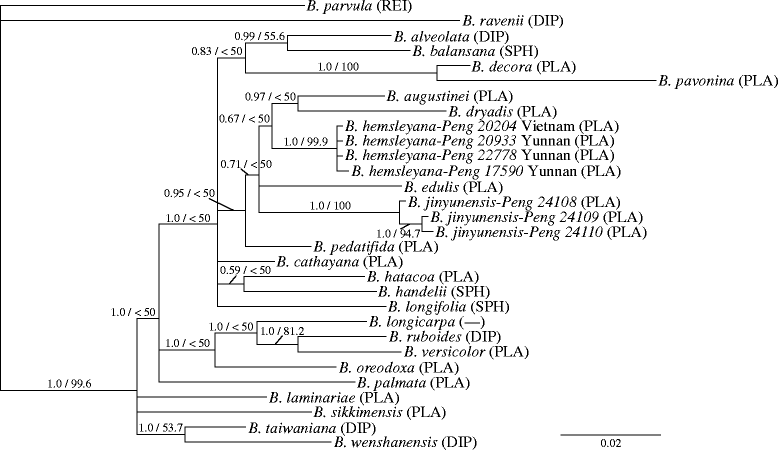

Supplement: Supplementary file 2 — Authors’ original file for figure 2 [file 40529_2014_9062_MOESM2_ESM.gif]

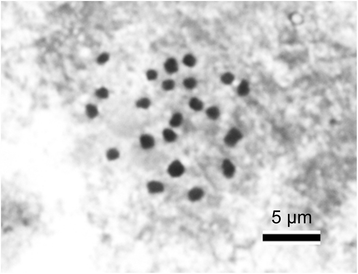

Supplement: Supplementary file 3 — Authors’ original file for figure 3 [file 40529_2014_9062_MOESM3_ESM.gif]

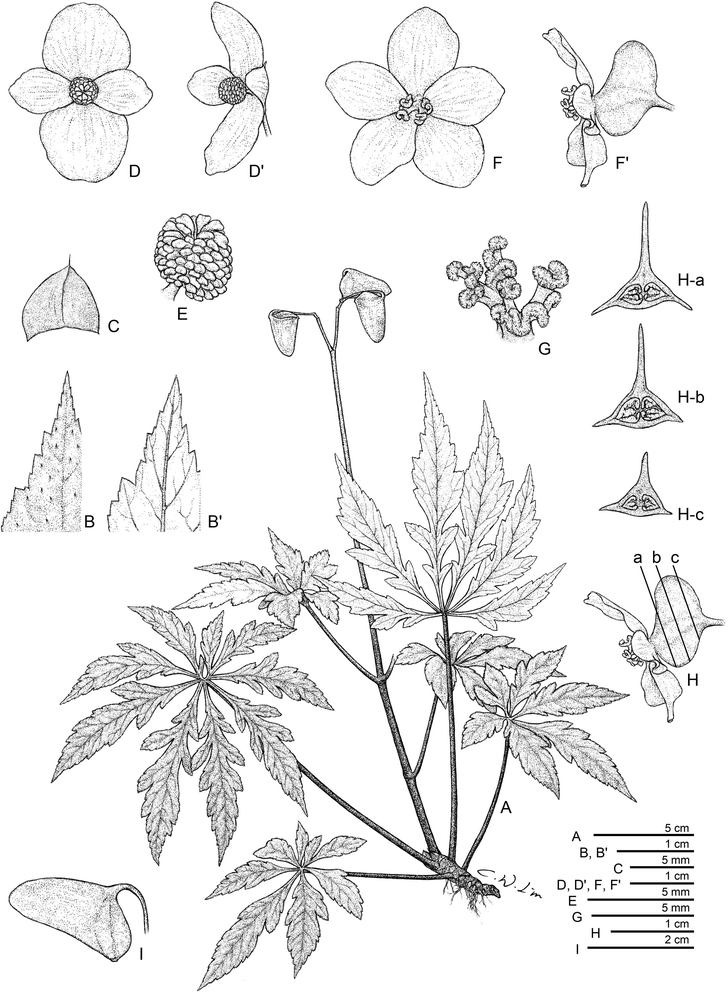

Supplement: Supplementary file 4 — Authors’ original file for figure 4 [file 40529_2014_9062_MOESM4_ESM.gif]

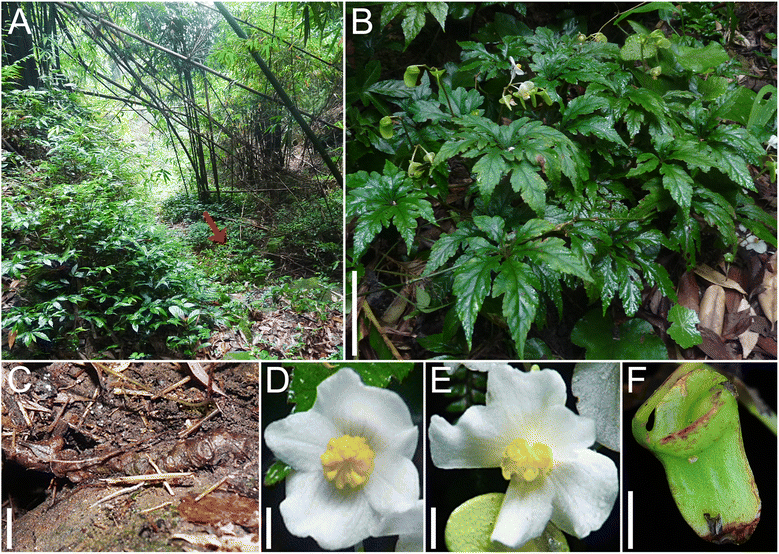

Supplement: Supplementary file 5 — Authors’ original file for figure 5 [file 40529_2014_9062_MOESM5_ESM.gif]
